# Hints for the Treatment of Suddenly-Developed Insanity

**Published:** 1898-06-25

**Authors:** F. St. John Bullen

**Affiliations:** Late Assistant Medical Officer and Pathologist, West Riding Asylum


### Medical Progress and Hospital Clinics.

[*The Editor will be glad to receive offers of co-operation and contributions from members of the profession. All letters should be addressed to* The Editor, at the Office, 28 & 29, Southampton Street, Strand, London, W.C.]

### HINTS FOR THE TREATMENT OF SUDDENLY-DEVELOPED INSANITY.

By F. St. John Bullen, M.R.C.S., Late Assistant Medical Officer and Pathologist, West Riding Asylum.

(*Concluded from page* 200).

As soon as a lapse in the excitement occurs, attempt should be made to administer nourishment, and if after several hours (a period varying with the physical condition) the symptoms continue unchanged, this must be effected by nasal or œsophageal tube rather than run any risk of collapse. The earlier the food be given, and the larger its quantity, the better the chance of a quick and complete recovery. The feed should consist of two eggs beaten up with one pint of hot milk and two ounces of port wine, or a smaller quantity of spirit. Early feeding is especially necessary in cases of melancholic type with feeble frame, and if a sedative is tried in these cases it is well to essay morphia given hypodermically. Therapeutic treatment will vary somewhat according to the conditions to which the frenzy is added. It will be best to enumerate a few drugs, with their more especial applicability.

**Chloral Hydrate and Bromide of Potash.**—These are the most appropriate drugs for epileptic subjects, and the first dose given may be half a drachm of the former and a drachm of the latter (provided no physical condition counter-indicates). A combination of these is also serviceable in acute alcoholic mania, alcoholic convulsions, delirium tremens (in moderate doses), and may be tried in frenzy of melancholic type. It is well to remember that the joint action of alcohol and chloral is more speedy and profound than that of the latter drug alone, and that alcohol does not serve to counteract the depressant qualities of chloral. The writer is in the habit of prescribing two drops of liq. atropiæ with the chloral and alcohol when given in considerable doses.

**Hyoscine.**—This for emergency purposes is best exhibited hypodermically. It can be used in all forms of mania, but from the writer's experience is almost useless in epileptic furor. A small dosage should always be used at first, a few persons being highly susceptible to it; once the patient is found normally tolerant, considerable doses can be safely administered. 1-100th grain is sufficient to commence with. Its use is said not to be counter-indicated by renal or cardiac disease.

**Paraldehyde.**—This is a most useful and safe drug in any form of mental perturbation, and has practically no counter-indications. It may be given from the first in doses of one or two drachms, and increased if necessary. With infusion of senega it forms a good emulsion. The same remarks as to use and efficacy apply to chloralamide; this, too, is free from the nauseous taste and odour of paraldehyde.

**Sulphonal.**—This is unsuitable for acute cases. Pot. bromide, tr. cannabis indica and hyoscine form a very efficacious combination, which may be tried in cases of noisy excitement. The B.P. doses of cannabis are useless, and half a drachm of the tincture may be used from the first; tr. hyoscyami, one or two drachms, can replace the hyoscine, if more easily obtained.

In cases of acute hysterical excitement drugs will probably be less serviceable than the cold douche. The water should be poured from a height of several feet upon the subject's head and face.

Some points which may be born in mind in the diagnosis of an acute attack of insanity are here briefly set forth; it is important to arrive at an opinion as to the probable duration of the case so as to decide whether removal from home must be at once undertaken.

**Age.**—Acute transient mental disturbance is not likely to be met with in persons of or beyond middle life where no previous attacks have occurred, but when so happening it is prodromal of serious organic disease. Mania-transitoria is to be expected rather amongst pubescents, adolescents, and younger adults.

**Cause, Onset, and Heredity.**—The less adequate the exciting cause, the more rapidly supervening the insanity, the more delirious in type the symptoms, and the more marked the hereditary predisposition, the greater the chance of the attack proving transient. Even without the heredity a similar prognosis could be given.

When the attack occurs during, or shortly after childbirth, or after a physical or moral shock, a recent apoplexy, acute rheumatism, pneumonia, or other inflammatory affection, chorea, in an epileptic person, or in one predisposed by heredity who has been drinking to excess, or has been suddenly cut off from an indulgence in alkaloids, there is a probability of its duration being short.

On the contrary, the older the patient, the longer the application of the suspected cause (be it alcohol, drugs, drain of lactation, &c.), the less delirious the character of symptoms, and especially the more the indication of physical decay, the greater the expectation of a prolonged attack. If the thermometer be called into use in diagnosis, which is always advisable, it should be borne in mind that a temperature over 100 Fahr. is unusual in uncomplicated mental disorder.

In case the immediate sequestration of the patient in an asylum be necessary from the outset this will be effected by means of an "urgency order." A brief reminder of its requirements is here appended: One medical certificate is alone required, together with an "order" from the person taking action in the matter; the latter and the certifier must each have seen the patient within two clear days from the date of signature of their respective documents. The order lasts (at present) for seven days, or whilst a petition is pending. The form of the urgency order is attached, in most cases, to the usual medical certificates. For the longer retention of the patient the ordinary form of procedure has to be carried out.

